# The Mediterranean Diet in the Stroke Belt: A Cross-Sectional Study on Adherence and Perceived Knowledge, Barriers, and Benefits

**DOI:** 10.3390/nu11081847

**Published:** 2019-08-09

**Authors:** Caroline J. Knight, Olivia Jackson, Imran Rahman, Donna O. Burnett, Andrew D. Frugé, Michael W. Greene

**Affiliations:** 1 Department of Nutrition, Dietetics, and Hospitality Management, Auburn University, Auburn, AL 36849, USA; 2 Boshell Diabetes and Metabolic Diseases Research Program, Auburn University, Auburn, AL 36849, USA

**Keywords:** Mediterranean diet, adherence, barriers and benefits, Stroke Belt, stages of change

## Abstract

The Mediterranean diet (MedDiet) is recommended by the current Dietary Guidelines for Americans, yet little is known about the perceived barriers and benefits to the diet in the U.S., particularly in the Stroke Belt (SB). Thus, the purpose of this study was to examine MedDiet adherence and perceived knowledge, benefits, and barriers to the MedDiet in the U.S. A cross-sectional study was conducted on 1447 participants in the U.S., and responses were sorted into geographic groups: the SB, California (CA), and all other US states (OtherUS). Linear models and multivariable linear regression analysis was used for data analysis. Convenience, sensory factors, and health were greater barriers to the MedDiet in the SB group, but not the OtherUS group (*p* < 0.05). Weight loss was considered a benefit of the MedDiet in the SB (*p* < 0.05), while price and familiarity were found to be less of a benefit (*p* < 0.05). Respondents with a bachelor’s degree or greater education had greater total MEDAS scores (*p* < 0.05) and obese participants had a lower MedDiet adherence score (*p* < 0.05). Our results identify key barriers and benefits of the MedDiet in the SB which can inform targeted MedDiet intervention studies.

## 1. Introduction

A traditional Mediterranean-based diet contains high intakes of fruit, vegetables, nuts, and whole grain cereals [1]. Red and processed meats, dairy products, and sweets are limited, while fish and poultry are encouraged in moderation. Extra virgin olive oil is the primary dietary fat, and red wine is preferred in moderation with meals, respecting social beliefs. The Mediterranean diet (MedDiet) was first identified as one of the healthiest patterns of eating in the Seven Countries Study: when extra virgin olive oil was the primary dietary fat, all-cause and coronary heart disease death rates were lowest [2]. More recently, it has been observed that adherence to a MedDiet is associated with reduced cardiovascular disease risk [3] and overall mortality [4], prevention and control of type II diabetes [5], and decreased risk of developing metabolic syndrome [6]. The MedDiet was added to the 2015 Dietary Guidelines for Americans as a recommended healthy food pattern to increase overall health [7]. More recently, the MedDiet was named the Best Diet of 2019 by U.S. News & World Report [8] and has been promoted by the American Heart Association for reduction of stroke risk [9] for its similar benefits to the DASH diet [10], while the American Diabetes Association [11,12] recommends a MedDiet for the prevention and treatment of type II Diabetes [13,14].

Theories and models of behavior change have been proposed to explain social determinates of health to increase nutrition education efficacy and encourage the adoption of healthy behaviors and diets. The Precaution Adoption Process Model (PAPM) is derived from the Transtheoretical Model and proposes that there are seven states that an individual can be in when deciding to adopt a health behavior (“unaware”, “unengaged”, “deciding”, “decided no”, “decided yes”, “action”, and “maintenance”), and that these are influenced by beliefs, experiences, prior knowledge, and perceived benefits and barriers towards this behavior [15,16]. Perceived benefits and barriers towards adopting a diet are strong predictors of food choice and how likely an individual is to change their diet [17]. Importantly, nutrition education tailored to an individual’s stage of change can increase behavior change outcomes [18].

The MedDiet is recommended as a healthy dietary approach because adherence to the MedDiet is associated with reduced risk of chronic diseases such as stroke [19]. There are regional differences in the U.S. where the stroke death rates are more than 10% greater than the U.S. average [20,21]: the National Heart, Lung, and Blood Institute defined this 11 state region (Alabama, Arkansas, Georgia, Indiana, Kentucky, Louisiana, Mississippi, North Carolina, South Carolina, Tennessee, and Virginia) as the Stroke Belt (SB). These states also have increased rates of hypertension and diabetes compared to non-SB states [22,23]. Given that the MedDiet is recommended as a healthy dietary approach to reduce the risk of stroke, it is surprising that little is known about factors associated with MedDiet adherence in the SB. In particular, there is currently no knowledge of perceived benefits or barriers towards the MedDiet or the stage of change towards adopting a MedDiet in the SB. The purpose of the present study was to assess in the SB: (1) MedDiet adherence; (2) perceived benefits and barriers towards a MedDiet; and (3) participants’ stage of change towards adopting a MedDiet.

## 2. Materials and Methods

### 2.1. Survey Instrument Development

A survey was developed to assess MedDiet adherence, participants’ stage of change towards adopting the MedDiet, perceived benefits and barriers of the MedDiet, and demographic variables. MedDiet adherence was evaluated using a validated 14-question Mediterranean Diet Adherence Screener (MEDAS) [24] that has been used to assess MedDiet adherence in countries bordering the Mediterranean Sea basin and elsewhere in the world, including the southeast U.S. [25,26,27,28] (Supplementary Materials Table S1). Three questions were asked to assess participants’ readiness to adopt a MedDiet using the Precaution Adoption Process Model (stages of change) [16] (Supplementary Materials Table S2). A pool of 100 questions measuring perceived benefits and barriers of the MedDiet was created by adapting questions from previously validated studies [29,30,31,32,33,34,35,36], assessing perceived benefits and barriers towards adopting a healthy diet. The questions were then screened by five registered dietitians to narrow down the questions by removing redundant and unclear questions to give a final 18 questions assessing perceived barriers to the MedDiet (knowledge, convenience, sensory appeal, and health; Supplementary Materials Table S3) and 26 questions assessing perceived benefits (knowledge, weight loss, ethical concerns, natural content, familiarity, price, sensory appeal, and mood; Supplementary Materials Table S4) that were measured using a five-point Likert scale. One question from the Health barrier was removed due to poor internal validity of the Health factor. Seven demographic and anthropometric questions determining age, sex, ethnicity, height, weight, level of education, and previous nutrition education or knowledge were assessed (Supplementary Materials Table S5). Body Mass Index (BMI) was calculated by dividing weight in pounds (lb) by height in inches (in) squared and multiplying by a conversion factor of 703. The Black-African and Black-Other categories were combined into the Black category due to only 12 participants being in the Black-Other category. Indian, Pakistani, and Asian-Other categories were also combined into the Asian-Other category due to only 28, 2, and 80 participants being in these categories, respectively. The Middle School education category was added to the High School Diploma category in our final analysis to create the High School or less category. 

### 2.2. Survey Distribution

This study was approved by the Auburn University institutional review board prior to distributing the surveys. This survey was distributed using Amazon Mechanical Turk (MTurk) from 9 September–14 November 2018. MTurk is an online platform that connects “requesters” with “workers” who perform an online task for the requester and receive compensation for it. Tasks are then either accepted or rejected if directions dictated by the requester are not followed. For this survey, workers were instructed that they must be located within the U.S. to participate. Separate projects were disseminated in MTurk to capture regional responses. Workers were compensated 0.60 US$ if the survey was accepted. Participants were eligible to complete the study if they were adults (≥18 years old) and had an approval rating greater than 90% for all previous MTurk survey responses. Workers were redirected to Qualtrics XM to complete the survey. The participants’ latitude and longitude positions were used to ensure participants were located in the U.S. before approving their response. The raw data was independently reviewed (DB) to ensure accuracy.

### 2.3. MedDiet Mapping

Survey data were downloaded from Qualtrics XM with latitude and longitude information for use in mapping MedDiet total scores. Batch reverse geocoding using services provided on the Texas A&M Geoservices Website was undertaken to provide the county, state, and zip code identification of respondents for use in statistical analyses. MedDiet total scores were visualized on a heat map created with ArcGIS^®^ software by Esri displayed with values ranging from 0 (low) to 13 (high); the color ramp increases in color depth (yellow to purple) as scores increase in value, as indicated on the map legend. The map was constructed using the Esri Canvas Base map, and the Esri Canvas Reference and USA States (Generalized) feature layers with data provided by Esri, HERE, Garmin, NGA (National Geospatial-Intelligence Agency), USGS (U.S. Geological Survey), and the National Geographic Society. The abbreviations for names of U.S. states were added to the map with Adobe Photoshop.

### 2.4. Statistical Analysis

IBM SPSS Statistics 23 was used to determine frequency distributions in the demographic data and perform Pearson’s chi-squared tests to analyze differences in demographic categories between groups and participants by stage of change. Multivariable linear regression, multivariate linear model, and logistic regression statistical analyses were conducted in R v3.52. A multivariable linear regression analysis was used to assess the differences in total MedDiet adherence scores between the groups adjusted for all covariates. A multivariate linear model was used to assess barrier and benefit question scores in the groups. Model 1 was unadjusted, Model 2 was adjusted for sex and age, and Model 3 was adjusted for all demographic variables. A backward stepwise logistic regression was performed to identify the predictors of the stage of change with the demographic variables. Inclusion and retention criteria in the logistic regression model were set at *p*-value cutoff points of 0.25 and 0.10, respectively. Akaike’s Information Criteria (AIC) was used to select the most parsimonious model. 

## 3. Results

### 3.1. Participants

**Figure 1:**
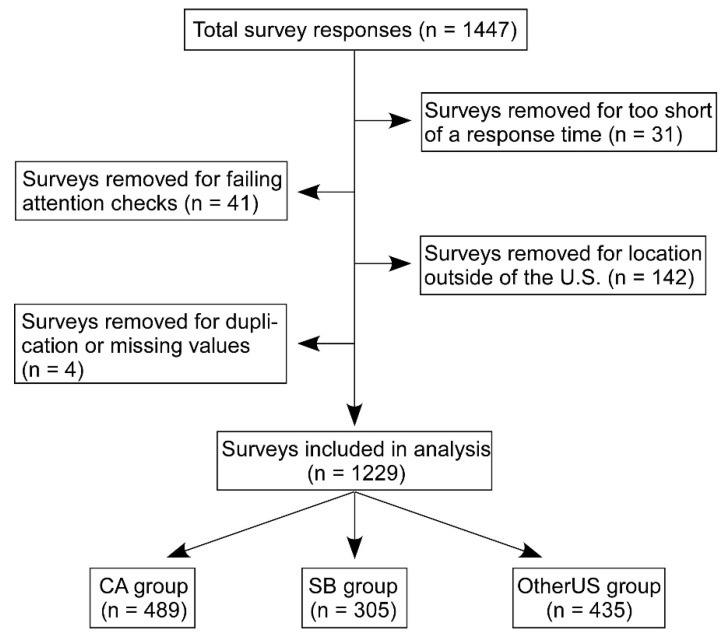
Survey responses were collected through Amazon Mechanical Turk. A total of 1443 responses were collected, and 31 were removed for completing the survey in less than 90 s, 41 were removed for failing to pass attention check questions located within the survey, and 142 surveys were rejected for not being located within the US. A total of 1229 surveys were used for analysis, with 489 from California (CA), 305 from the Stroke Belt (SB), and 435 from other locations within the United States (OtherUS).

The survey was completed by 1447 participants (Figure 1). After exclusion for: (1) the survey being completed in less than 90 s (*n* = 31); (2) the participant failing to pass two of the three attention check questions in the survey (*n* = 41); (3) the participant being located outside of the United States (*n* = 142); or (4) having a duplicate response or missing values (*n* = 4), 1229 valid responses were obtained. The responses were then sorted into three groups based on geographic location: California (CA) (*n* = 489), Stroke Belt (SB) (*n* = 305), and OtherUS (*n* = 435), and the CA respondents served as the reference group. California was selected as the reference group due to its Mediterranean climate [37,38] and recent data showing it is a hotspot for adherence to the Mediterranean diet in the U.S. [39].

### 3.2. Demographics

We first determined whether there were demographic differences between participants in the CA, SB, and OtherUS groups. As shown in Table 1, the SB group had a greater proportion of females, older (>55 years old) participants, and obese participants. In contrast, the CA group had the greatest proportion of the youngest (18–24 years old) participants and non-white participants. There were no differences between groups in previous nutrition education.

### 3.3. Mediterranean Diet Adherence

**Figure 2:**
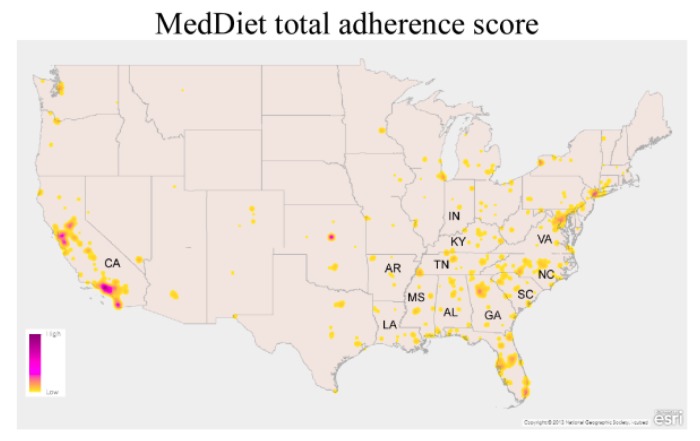
Heat map of MedDiet total adherence score data (low = 0; high = 13) created with ArcGIS^®^ software by Esri; color ramp increases in color depth (yellow to purple) as scores increase in value as indicated on the map legend. Source data: Esri, HERE, Garmin, NGA (National Geospatial-Intelligence Agency), USGS (U.S. Geological Survey), and the National Geographic Society. California and states in the Stroke Belt are indicated by their two-letter abbreviations.

The total MedDiet adherence screener (MEDAS) score was analyzed using multivariable linear regression adjusting for demographic variables, stages of change, barriers, and benefits and was found to be lower in the SB and OtherUS groups in comparison to the CA group (Table 2). For each point increase in MEDAS score in the CA group, a reduction in 0.31 ± 0.16 points (*p* = 0.047) and 0.47 ± 0.14 points (*p* < 0.001) was observed in the SB and OtherUS groups, respectively. Consistent with these findings we observed that when MEDAS scores were plotted on a map of the U.S., high MEDAS scores (represented as purple) were concentrated in CA, while low MEDAS scores (represented as yellow) were observed in the SB (Figure 2). In our assessment of demographic variables and MEDAS score, we observed that MEDAS scores were increased 0.36 ± 0.17 points in those with a bachelor’s degree (*p* = 0.032) and 0.48 ± 0.21 points in those with a master’s degree (*p* = 0.022). Obese participants had lower MEDAS scores by 0.36 ± 0.15 points (*p* = 0.019). Participants in the Action/Maintenance stage of change had greater MEDAS scores by 0.48 ± 0.18 points (*p* = 0.008). An increase in Knowledge barrier score was associated with a 0.10 ± 0.03 increase in MEDAS score (*p* < 0.001), while a greater Sensory Appeal score led to an increase in MEDAS scores of 0.18 ± 0.03 (*p* < 0.001). For the benefit factors, an increase in Familiarity score led to a 0.12 ± 0.03 (*p* < 0.001) increase in MEDAS scores. Increased Sensory Appeal scores for the MedDiet also led to increased MEDAS scores by 0.13 ± 0.05 (*p* = 0.006). There were no differences in sex, age, ethnicity, or nutrition qualifications. 

### 3.4. Barriers to Consuming a MedDiet

Perceived barriers to adopting a MedDiet were measured using 18 questions that were sorted a priori into four factors: Knowledge, Convenience, Sensory Appeal, and Health (Supplementary Materials Table S3). To assess internal consistency of the questions in the four barrier factors, Cronbach’s alpha was calculated: while values above 0.70 are considered to be best for determining internal validity, above 0.60 is considered acceptable or adequate [40,41,42,43]. The Knowledge barrier had a Cronbach’s α = 0.429, indicating poor reliability for the questions in this factor (Table 3). Removal of individual questions did not improve the reliability of the knowledge factor (data not shown). The Convenience (Cronbach’s α = 0.725) and Sensory Appeal (Cronbach’s α = 0.701) barriers had acceptable reliability. In the Health barrier, one question was removed from the analysis to improve Cronbach’s α from 0.663 to 0.778 (Supplementary Materials Table S3).

We used a linear regression model that was unadjusted (Model 1), adjusted for sex and age (Model 2), and adjusted for sex, age, ethnicity, education, and BMI (Model 3) to assess knowledge, convenience, sensory appeal, and health barriers in the SB and OtherUS groups using the CA group as a reference. All four of the barriers, knowledge (β = 0.569, SE = 0.212, *p* = 0.007) convenience (β = 0.955, SE = 0.251, *p* = <0.001), sensory appeal (β = 0.650, SE = 0.202, *p* = 0.001), and health (β = 0.981, SE = 0.217, *p* = <0.001) were observed to be greater barriers to the MedDiet in the SB group in Model 3 compared to the CA group (Table 3). This relationship was maintained in Models 1 and 2. In the OtherUS group, knowledge was also a greater barrier in Model 3 (β = 0.387, SE = 0.190, *p* = 0.042), as was convenience (β = 0.466, SE = 0.225, *p* = 0.038) when compared to the CA group: these findings were also observed in Models 1 and 2.

### 3.5. Benefits of Consuming a MedDiet

Perceived benefits from adopting a MedDiet were measured using 26 questions that were sorted a priori into eight factors: Health, Weight Loss, Ethical Concerns, Natural Content, Familiarity, Price, Sensory Appeal, and Mood (Supplementary Materials Table S4). Internal consistency was calculated for these factors using Cronbach’s α (Health = 0.857; Weight Loss = 0.635; Natural Content 0.610; Ethical Concerns = 0.801; Familiarity = 0.619; Price = 0.719; Sensory Appeal = 0.618; Mood = 0.795). A linear regression model that was unadjusted (Model 1), adjusted for sex and age (Model 2), and adjusted for sex, age, ethnicity, education, and BMI (Model 3) was used to assess the benefits from adopting a MedDiet in the SB and OtherUS groups using the CA group as a reference (Table 4). The SB group considered the MedDiet to be much more likely to produce weight loss in Model 3 (β = 0.333, SE = 0.121, *p* = 0.006), and this association was consistent in Models 1 and 2. Familiarity (β = −0.554, SE = 0.156, *p* < 0.001) and Price (β = −0.352, SE = 0.152, *p* = 0.021) were both considered less of a benefit of the MedDiet in the SB group in Model 3, as well as in Models 1 and 2. The OtherUS group had no differences from the CA group.

### 3.6. Stages of Change & Demographic Influences

The CA group had a greater number of participants in the Decided Yes category while the OtherUS group had fewer than the SB group (*p* < 0.05) (Table 5). The OtherUS group also had more participants in the Action/Maintenance stage (*p* < 0.05). There was no difference between groups in percentage of participants in the Unaware/Unengaged, Deciding, or Decided No stages of change. There was also no difference between groups in having heard of the MedDiet before (data not shown).

Logistic regression was performed to determine the effect of demographic variables on likelihood to be in each stage of change towards adopting the MedDiet (Table 6, Table 7 and Table 8). Participants were less likely to be in the Unengaged/Unaware stage in the CA group if they had any education greater than a high school education (*p* < 0.05). Chinese participants in the CA and OtherUS groups were more likely to be in the Unaware/Unengaged stage, as well as Asian-other and Other respondents in the CA group (*p* < 0.05). 

Those with higher education in the CA group were at least two times more likely (OR = 2.75, 95% CI: 1.18–6.65) to be in the Action/Maintenance stage (*p* < 0.05), and Black participants in the CA and OtherUS groups had greater odds of being in this stage as well (*p* < 0.05). There was reduced odds (OR = 0.31, 95% CI: 0.14–0.61) for obese participants to be in the Action/Maintenance phase in the CA group. In OtherUS participants who were between the ages of 35–44, there was a reduced likelihood of being in the Action/Maintenance stage (OR = 0.54, 95% CI: 0.30–0.94).

The OtherUS was the only group with demographic factors that influenced the Decided No stage. Those who were Chinese or Other ethnicities were 5.04 (95% CI: 0.73–21.73) and 3.95 (95% CI = 0.86–13.40) times more likely to have decided not to eat a MedDiet (*p* < 0.05), and were 6.48 (95% CI = 0.92–29.58) times more likely to be in the Decided No stage if they had a GED (*p* < 0.05). Participants in the CA group had greater odds of being in the Deciding stage if they were overweight (*p* < 0.01), and were less likely if they were female or Chinese. Black participants were also less likely to be in the Deciding stage in the OtherUS group (OR = 0.38, 95% CI: 0.17–0.75). In the SB group, obese participants were 5.46 (95% CI = 2.08–16.23) times more likely to be in the Decided Yes stage (*p* < 0.01). The CA group participants had greater odds of being in the Decided Yes stage if they were 35–44 years old, 55–64 years old, or obese (*p* < 0.05), while participants in the OtherUS group had increased odds (OR = 2.77, 95% CI = 1.41–5.68) for being female.

## 4. Discussion

MedDiet adherence and factors influencing adherence has not previously been measured across geographical regions of the U.S. Therefore, we developed a survey instrument to assess MedDiet adherence, perceived benefits and barriers of the MedDiet, and stage of change towards adopting the MedDiet. In the present study, the majority of respondents had at least an associate’s degree, and there were no differences between groups in relation to education, which has been associated with nutrition knowledge and adherence [44]. MTurk worker populations have been shown to be more diverse than typical student or internet samples, without any significant differences in the quality of the data [45,46]. The survey utilized multiple practices suggested by Kees et al. for high-quality MTurk data [46], including utilizing location to check respondents’ locations, specifying a required previous acceptance rate of at least 90%, offering greater compensation than other surveys, including three attention checks throughout the survey, and implementing a minimum time requirement. MTurk worker demographics are typically male, younger, have higher education, and make less money than a true representative sample of the U.S.; yet, the population in the present study had a greater percentage of female respondents than previously reported in MTurk populations [45,46,47].

Geographical differences in MedDiet adherence within countries (Italy and Spain) in the Mediterranean Basin have been observed [48,49,50,51]. Consistent with these findings, we observed geographical differences in MedDiet adherence in the US: MedDiet adherence scores were lower in both the SB and OtherUS groups compared to the CA group. Our findings are consistent with the observation that CA has recently been identified as a hot spot for MedDiet adherence while the southeast US was identified as a cold spot [39]. Regional differences in stroke risk between California and the SB have also been observed. The CARDIA study found over a 7-year period that participants in Oakland, CA had significantly lower BP than those in Birmingham, AL and concluded that elevated blood pressure in AL is a contributing factor due to its position in the SB [52]. Our findings of lower MedDiet adherence in the SB could be contributing to the elevated blood pressure. However, formal testing of the link between high blood pressure and low MedDiet adherence in the SB is required. The lower MedDiet adherence scores in the SB are also consistent with the observation that the MedDiet is effective for maintaining a healthy weight [12], and that the rate of obesity in CA is the 2nd lowest in the U.S., while the prevalence of obesity in the SB is significantly greater than the rest of the U.S. [53]. Our results demonstrating that obese participants had lower MedDiet adherence are consistent with those reported for obese adults in Spain [54] and children in Italy [50].

We found that participants with bachelor’s or master’s degrees were more likely to follow a MedDiet compared to those with lower education, which confirms previous results correlating greater education with more willingness to adopt a healthy diet in Spain [44] and with MedDiet adherence in Alabama [28] and cardiology patients in Oklahoma [55]. Increases in perceived Knowledge and Sensory Appeal barriers were associated with an increase in MedDiet score. Furthermore, perceiving Familiarity and Sensory Appeal as greater benefits was associated with increased MedDiet adherence scores.

All four perceived barriers (Knowledge, Convenience, Sensory Appeal, and Health) were considered significant barriers to the MedDiet in the SB group compared to the CA group when adjusted for all demographic factors. Our results demonstrate that regional differences in barriers to the MedDiet exist in the U.S. Previous examinations of barriers towards adopting a healthy diet in Spain and Europe found that sensory appeal, knowledge, and convenience were also significant factors for reluctance of individuals to adopt a healthy diet [44]; and Pitts et al. identified access and convenience as the primary roadblock towards adopting healthier diets in the SB [56]. The perceived Health barrier had the greatest decrease in the SB group compared to CA group, specifying that the participants in this SB group considered the MedDiet to be unhealthy. These results suggest that participants in the SB are misinformed on the health benefits of the MedDiet. Knowledge of the MedDiet is a unique barrier to the MedDiet in the U.S., as we observed in the SB and OtherUS groups, there is a lack of understanding about the diet itself that countries surrounding the Mediterranean Sea do not share. Convenience was also a greater barrier towards the MedDiet in the OtherUS group, indicating that outside of CA, the MedDiet is considered as being inconvenient to follow. Indeed, commodity organizations in CA are actively promoting the MedDiet by marketing it as an easy, healthful way to eat [37] which could be influencing the perceived barrier Convenience for participants living in CA compared to the SB.

The perceived benefits, Price and Familiarity, were seen as less of a benefit of the MedDiet in the SB group compared to the CA group. However, Weight Loss was seen as more of a benefit in the SB group than in the CA group. This indicates that in the SB group, the MedDiet is possibly considered more of a weight loss diet as opposed to a healthy lifestyle. While Price and Familiarity are expected concerns in this area due to a lack of knowledge and marketing of the MedDiet, Weight Loss is a unique factor that should be evaluated further to determine if the current MedDiet promotion in this area is skewed towards weight loss instead of marketing it as a healthful lifestyle change. The OtherUS group had no differences in perceived benefits in comparison to the CA group, indicating that these factors are unique to the SB group.

Logistic regression analysis of stage of change suggested that in the CA group, those with any level of education greater than a General Education Diploma (GED) were at least two times more likely to be in the Action/Maintenance stage and had a lower OR of being in the Unaware/Unengaged stage. These results are consistent with previous studies showing a correlation between education and MedDiet adherence [28,55,57]. Further, education is also correlated with nutrition knowledge [28,58,59,60,61,62]. Obese individuals in the CA group were also less likely to be in the Action/Maintenance stage, indicating that education and weight status are indicators of stage of change towards adopting a MedDiet diet. Those who have lower levels of education are more likely to be in the Unaware/Unengaged stage in the CA group. Understanding the role of demographic factors in stage of change towards adopting a MedDiet can improve nutrition education efforts in those populations. Previous studies have shown that when nutrition education is targeted towards a person’s stage of change, it is more likely to result in the goal behavior change [15].

The only self-reported variable that played a role in participant’s stage of change was BMI in the SB group. Obese participants were approximately five times more likely to be in the Decided Yes stage. When taken into account with the data from the MedDiet adherence scores, this confirms that geographic location (the SB) impacts both MedDiet adherence and stage of change towards adopting the MedDiet.

OtherUS participants were six times more likely to be in the Decided No category if the participant had a GED, confirming previous findings that those with lower education were less likely to follow a MedDiet [28,55,57].

This survey was most notably limited by the MTurk population. While there were no significant differences between groups that are believed to have influenced the results, the MTurk population is not representative of the U.S. Participants’ locations for grouping was determined by latitude and longitude; however, it is possible that respondents were traveling or not native to the location where the survey was completed which could lead to them being incorrectly sorted into a group. Stage of change and MedDiet adherence data were subjective and could be influenced by self-selection into the study or personal bias.

## 5. Conclusions

This study identifies key barriers and benefits of the MedDiet in the SB which can inform future targeted MedDiet intervention studies. Specifically, our data suggest that future nutrition education interventions should be aimed at improving knowledge about the MedDiet and its health benefits and ways to reduce barriers to consuming the MedDiet in the SB.

## Figures and Tables

**Table 1:** Demographics of participants by geographic groups.

Characteristic	CA ^†^ (*n* = 489)		SB (*n* = 305)			OtherUS (*n* = 435)	
	*n*	%	*n*	%	*n*	%	*p*-value
Sex *							<0.001
Male	214	43.8	107	35.1	228	52.4	
Female	275	56.2	198	64.9	207	47.6	
Age *							0.009
18–24	74	15.1	30	9.8	36	8.3	
25–34	203	41.5	121	39.7	190	43.7	
35–44	110	22.5	69	22.6	103	23.7	
45–54	59	12.1	33	10.8	58	13.3	
55–64	30	6.1	37	12.1	34	7.8	
>65	13	2.7	15	4.9	14	3.2	
Ethnicity *							<0.001
White	285	58.3	236	77.4	333	76.6	
Black	31	6.3	47	15.4	53	12.2	
Chinese	52	10.6	3	1.0	11	2.5	
Asian-other	79	16.2	12	3.9	19	4.4	
Other ethnic group	42	8.6	7	2.3	19	4.3	
Education							0.178
High School or lower	83	17.0	59	19.3	56	12.8	
GED ^‡^	11	2.2	9	3.0	9	2.1	
Technical or trade certificate	31	6.3	23	7.5	20	4.6	
Associate degree	65	13.3	47	15.4	60	13.8	
Bachelor’s degree	229	46.8	127	41.6	215	49.4	
Master’s or professional degree	70	14.3	40	13.1	75	17.2	
BMI *							<0.001
Underweight	22	4.5	3	1.0	12	2.8	
Normal weight	241	49.3	112	36.7	204	46.9	
Overweight	129	26.4	105	34.4	133	30.6	
Obese	96	19.6	82	26.9	80	18.4	
Unknown	1	0.2	3	1.0	6	1.4	
Health or nutrition related qualifications							0.834
Yes	24	4.9	13	4.3	18	4.1	
No	465	95.1	292	95.7	417	95.9	

* Significance across score categories by Pearson’s chi-squared test. ^†^ CA, California; SB, Stroke Belt; OtherUS, other U.S. states. ^‡^ GED, General Education Diploma.

**Table 2:** Multivariable linear regression analysis assessing Mediterranean diet adherence between groups adjusted for demographic categories, stages of change, barriers, and benefits.

Characteristic		β	SE	*p*-Value
Group	CA ^†^	Ref		
	SB	−0.309	0.155	0.047 *
	OtherUS	−0.468	0.138	**<0.001**
Sex	Male	Ref		
	Female	−0.034	0.117	0.773
Age	18–24	Ref		
	25–34	−0.003	0.192	0.990
	35–44	0.074	0.211	0.725
	45–54	0.190	0.239	0.427
	55–64	0.309	0.267	0.248
	>65	0.164	0.353	0.642
Ethnicity	White	Ref		
	Black All	0.007	0.190	0.972
	Chinese	−0.312	0.263	0.235
	Asian—Other	0.050	0.209	0.812
	Other	0.091	0.251	0.718
Education	High School or lower	Ref		
	GED ^‡^	0.103	0.391	0.787
	Technical Degree	0.100	0.270	0.711
	Associate’s Degree	0.326	0.208	0.118
	Bachelor’s Degree	0.357	0.167	**0.032**
	Master’s Degree	0.475	0.207	**0.022**
BMI	Healthy	Ref		
	Under	0.260	0.337	0.441
	Overweight	−0.034	0.137	0.802
	Obese	−0.358	0.153	**0.019**
	Unknown	0.445	0.632	0.482
Nutrition Qualification	No	Ref		
	Yes	0.038	0.274	0.890
Stage of Change	Unaware/Unengaged	Ref		
	Deciding	0.124	0.155	0.427
	Deciding No	−0.268	0.257	0.298
	Deciding Yes	0.049	0.205	0.810
	Action/Maintenance	0.476	0.179	**0.008**
Barriers	Knowledge	0.098	0.026	**<0.001**
	Convenience	0.019	0.024	0.437
	Sensory Appeal	0.176	0.033	**<0.001**
	Health	0.029	0.029	0.316
Benefits	Health	−0.006	0.018	0.728
	Weight Loss	−0.017	0.054	0.753
	Ethical	0.022	0.035	0.520
	Natural Content	0.051	0.055	0.355
	Familiarity	0.118	0.034	**<0.001**
	Price	0.070	0.036	0.054
	Sensory Appeal	0.126	0.046	**0.006**
	Mood	−0.020	0.031	0.521

* *p* values < 0.05 are indicated in bold font. ^†^ CA, California; SB, Stroke Belt; OtherUS, other U.S. states. ^‡^ GED, General Education Diploma.

**Table 3:** Multivariate linear analysis of MedDiet barriers across geographic groups.

Barriers	CA ^#^	SB			Other US		
		β	SE	*p*-value	β	SE	*p*-value
Knowledge (*n* = 4) ^‡^ (Cronbach’s Alpha = 0.429)							
Model 1 ^†^	Ref	0.590	0.201	0.003 *	0.434	0.181	**0.017**
Model 2 ^††^	Ref	0.556	0.202	**0.006**	0.400	0.182	**0.028**
Model 3 ^†††^	Ref	0.569	0.212	**0.007**	0.387	0.190	**0.042**
Convenience (*n* = 4) (Cronbach’s Alpha = 0.725)							
Model 1	Ref	0.984	0.240	**<0.001**	0.445	0.217	**0.040**
Model 2	Ref	0.903	0.240	**<0.001**	0.460	0.217	**0.034**
Model 3	Ref	0.955	0.251	**<0.001**	0.466	0.225	**0.038**
Sensory Appeal (*n* = 3) (Cronbach’s Alpha = 0.701)							
Model 1	Ref	0.702	0.193	**<0.001**	0.083	0.175	0.636
Model 2	Ref	0.628	0.193	**0.001**	0.099	0.174	0.571
Model 3	Ref	0.650	0.202	**0.001**	0.070	0.181	0.700
Health (*n* = 3) (Cronbach’s Alpha = 0.788)							
Model 1	Ref	0.893	0.111	**<0.001**	0.300	0.162	0.064
Model 2	Ref	0.816	0.178	**<0.001**	0.304	0.161	0.058
Model 3	Ref	0.852	0.186	**<0.001**	0.315	0.167	0.059

^‡^ Number of questions in each factor. * *p* values < 0.05 are indicated in bold font. ^†^ Model 1 (unadjusted). ^††^ Model 2 (adjusted for sex and age). ^†††^ Model 3 (adjusted for sex, age, ethnicity, education, and BMI). CA ^#^, California; SB, Stroke Belt; OtherUS, other U.S. states.

**Table 4:** Multivariate analysis of MD benefits across geographic groups.

Benefits	CA ^#^	SB			OtherUS		
		β	SE	*p*-value	β	SE	*p*-value
Health (*n* = 10) ^‡^ (Cronbach’s Alpha = 0.857)							
Model 1^†^	Ref	0.391	0.458	0.393	0.169	0.413	0.682
Model 2^††^	Ref	0.277	0.460	0.547	0.232	0.415	0.577
Model 3^†††^	Ref	0.360	0.482	0.455	0.230	0.432	0.594
Weight Loss (*n* = 2) (Cronbach’s Alpha = 0.635)							
Model 1	Ref	0.301	0.115	**0.009**	0.149	0.104	0.149
Model 2	Ref	0.284	0.116	**0.014**	0.152	0.104	0.145
Model 3	Ref	0.333	0.121	**0.006**	0.179	0.109	0.100
Ethical (*n* = 2) (Cronbach’s Alpha = 0.801)							
Model 1	Ref	−0.177	0.149	0.237	0.135	0.135	0.317
Model 2	Ref	−0.191	0.150	0.202	0.172	0.135	0.203
Model 3	Ref	−0.128	0.156	0.414	0.198	0.140	0.158
Natural Content (*n* = 2) (Cronbach’s Alpha = 0.610)							
Model 1	Ref	0.085	0.113	0.455	−0.133	0.104	0.194
Model 2	Ref	0.054	0.114	0.634	−0.122	0.103	0.234
Model 3	Ref	0.062	0.119	0.605	−0.131	0.107	0.222
Familiarity (*n* = 2) (Cronbach’s Alpha = 0.619)							
Model 1	Ref	−0.514	0.149	**<0.001**	0.123	0.135	0.362
Model 2	Ref	−0.504	0.150	**<0.001**	0.132	0.135	0.330
Model 3	Ref	−0.554	0.156	**<0.001**	0.044	0.140	0.752
Price (*n* = 2) (Cronbach’s Alpha = 0.719)							
Model 1	Ref	−0.352	0.144	**0.015**	−0.137	0.130	0.291
Model 2	Ref	−0.346	0.145	**0.017**	−0.135	0.131	0.302
Model 3	Ref	−0.352	0.152	**0.021**	−0.171	0.136	0.210
Sensory Appeal (*n* = 2) (Cronbach’s Alpha = 0.618)							
Model 1	Ref	0.026	0.117	0.823	−0.103	0.106	0.330
Model 2	Ref	−0.020	0.117	0.861	−0.117	0.106	0.267
Model 3	Ref	−0.060	0.123	0.624	−0.177	0.110	0.109
Mood (*n* = 3) (Cronbach’s Alpha = 0.795)							
Model 1	Ref	−0.206	0.198	0.299	−0.034	0.179	0.849
Model 2	Ref	−0.216	0.200	0.280	−0.044	0.181	0.807
Model 3	Ref	−0.136	0.210	0.515	−0.016	0.188	0.934

^‡^ Number of questions in each factor. * *p* values < 0.05 are indicated in bold font. ^†^ Model 1 (unadjusted). ^††^ Model 2 (adjusted for gender and age). ^†††^ Model 3 (adjusted for gender, age, ethnicity, education, and BMI). CA ^#^, California; SB, Stroke Belt; OtherUS, other U.S. states.

**Table 5:** Percent of participants in the CA, SB, and OtherUS groups by stage of change.

Stages of Change	CA ^#^	SB	OtherUS
Unaware/Unengaged	22.1	21.0	20.5
Deciding	35.4	40.0	36.6
Decided No	5.3	8.5	6.0
Decided Yes *	16.6	11.5	9.7
Action/Maintenance *	20.7	19.0	27.4

* Significance across score categories by Pearson’s chi-squared test (*p* < 0.05). CA ^#^, California; SB, Stroke Belt; OtherUS, other U.S. states.

**Table 6:** Backward stepwise logistic regression of stage of change by demographic factors in the CA group.

Characteristics			Stages of Change		
	Unaware/Unengaged	Deciding	Decided Yes	Decided No	Action/Maintenance
	OR (95% CI) ^‡^	OR (95% CI)	OR (95% CI)	OR (95% CI)	OR (95% CI)
Sex					
Female	-	0.67 (0.45–0.99) *	1.62 (0.98–2.75)	-	-
Age					
25–34	-	-	1.83 (0.95–3.70)	-	-
35–44	-	-	2.94 (1.44–6.22) **	0.28 (0.05–0.98)	-
45–54	-	1.73 (0.97–3.07)	-	-	-
55–64	-	-	5.65 (2.22–14.47) ***	-	-
Ethnicity					
Black	-	-	-	-	2.58 (1.03–6.15) *
Chinese	2.57 (1.28–5.06) **	0.35 (0.16–0.70) **	-	-	-
Asian-other	1.92 (1.05–3.41) *	-	-	-	-
Other	2.10 (0.99–4.28) *	-	-	-	-
Education					
Certificate	0.20 (0.04–0.63) *	-	-	-	3.01 (1.03–8.63) *
Associate’s	0.32 (0.14–0.69) **	-	-	-	3.44 (1.49–8.32) **
Bachelor’s	0.43 (0.25–0.74) **	-	-	2.18 (0.97–5.21)	2.08 (1.04–4.52) *
Master’s or professional	0.29 (0.13–0.63) **	-	-	-	2.75 (1.18–6.65) *
BMI					
Underweight	2.20 (0.81–5.58)	-	-	-	-
Overweight	-	1.89 (0.33–0.83) **	-	-	-
Obese	-	-	1.98 (1.12–3.43) *	-	0.31 (0.14–0.61) *

* *p*-value < 0.05; ** *p*-value < 0.01; *** *p*-value < 0.001. - Not applicable. ^‡^ Odds Ratio (OR); 95% Confidence Interval (CI).

**Table 7:** Backward stepwise logistic regression of stage of change by demographic factors in the SB group.

Characteristics			Stages of Change		
	Unaware/Unengaged	Deciding	Decided Yes	Decided No	Action/Maintenance
	OR (95% CI) ^‡^	OR (95% CI)	OR (95% CI)	OR (95% CI)	OR (95% CI)
Age					
35–44	-	-	-	0.33 (0.08–1.01)	-
45–54	-	-	-	0.23 (0.01–1.16)	-
55–64	-	-	1.85 (0.67–4.69)	0.42 (0.06–1.52)	-
Ethnicity					
Black	-	-	0.45 (0.10–1.40)	-	-
Chinese	11.05 (1.01–244.93)	-	-	-	-
Education					
Bachelor’s	0.57 (0.31–1.02)	-	-	-	1.52 (0.85–2.71)
BMI					
Overweight	-	-	2.31 (0.84–6.97)	-	-
Obese	-	0.68 (0.40–1.14)	5.46 (2.08–16.23) **	-	-
Health Qualifications					
Yes	-	-	-	-	2.70 (0.78–8.47)

* *p*-value < 0.05; ** *p*-value < 0.01; *** *p*-value < 0.001. - Not applicable. ^‡^ Odds Ratio (OR); 95% Confidence Interval (CI).

**Table 8:** Backward stepwise logistic regression of stage of change by demographic factors in the OtherUS group.

Characteristics		Stages of Change			
	Unaware/Unengaged	Deciding	Decided Yes	Decided No	Action/Maintenance
	OR (95% CI) ^‡^	OR (95% CI)	OR (95% CI)	OR (95% CI)	OR (95% CI)
Sex					
Female	-	-	2.77 (1.41–5.68) **	-	-
Age					
35–44	-	-	-	-	0.54 (0.30–0.94) *
45–54	-	-	0.14 (.01–0.66)	-	-
55–64	-	-	2.12 (0.77–5.21)	2.84 (0.78–8.32)	-
>65	-	2.93 (0.99–9.70)	-	-	0.20 (0.01–1.06)
Ethnicity					
Black	-	0.38 (0.17–0.75) **	-	-	4.18 (2.29–7.75) ***
Chinese	3.72 (1.03–12.87) *	-	-	5.04 (0.73–21.73) *	-
Other	-	-	-	3.95 (0.86–13.40) *	-
Education					
GED ^‡^	-	-	-	6.48 (0.92–29.58) *	-
Associate’s	-	-	-	-	2.20 (1.00–4.91)
Bachelor’s	0.71 (0.44–1.14)	-	-	-	1.58 (0.85–3.08)
Master’s or professional	-	-	-	-	2.03 (0.96–4.41)
BMI					
Underweight	-	-	-	-	2.07 (0.67–6.24)
Overweight	-	-	1.72 (0.84–3.43)	-	-
Obese	0.52 (0.25–1.00)	-	-	-	-

* *p*-value < 0.05; ** *p*-value < 0.01; *** *p*-value < 0.001. - Not applicable. ^‡^ Odds Ratio (OR); 95% Confidence Interval (CI). GED, General Education Diploma.

## Supplementary Materials

The following are available online at https://www.mdpi.com/2072-6643/11/8/1847/s1, Table S1: Mediterranean Diet Adherence Screener, Table S2: Stage of changes questions and benefits and barriers section, Table S3: Barrier Questions with Factors, Table S4: Benefit Questions with Factors, Table S5: Demographic and anthropomorphic questions.
