# Supplementary material for: The Mediterranean Diet in the Stroke Belt: A Cross-Sectional Study on Adherence and Perceived Knowledge, Barriers, and Benefits
Source: Nutrients. 2019 Aug 9;11(8):1847. doi: 10.3390/nu11081847 (PMC6723093; doi:10.3390/nu11081847)
Supplement: Supplementary file 1 [file nutrients-11-01847-s001.pdf]

Table S1: Mediterranean Diet Adherence Screener

We would like to ask you a few questions about your diet:

Question	Check the box that applies		
1. Do you use olive oil as main culinary fat?	Yes ☐	No ☐	
2. How many tablespoons of olive oil do you consume in a given day (including oil used for frying, salads, out-of-house meals, etc.)?	<1 ☐	1-4 ☐	>4 ☐
3. How many vegetable servings do you consume per day? (1 serving: ½ cup cooked, 1 cup raw [consider side dishes as half a serving])	<1 ☐	1-2 ☐	>2 ☐
4. How many fruit units (including natural fruit juices) do you consume per day? (1 serving: 1 cup)	<1 ☐	1-3 ☐	>3 ☐
5. How many servings of red meat, hamburger, or meat products (ham, sausage, etc.) do you consume per day? (1 serving: 2-3 ounces)	<1 ☐	1-3 ☐	>3 ☐
6. How many servings of butter, margarine, or cream do you consume per day? (1 serving: 1 tablespoon)	<1 ☐	1-3 ☐	>3 ☐
7. How many sweet or carbonated beverages do you drink per day?	<1 ☐	1-3 ☐	>3 ☐
8. How many glasses of wine do you drink per week? ☐Red ☐White ☐Both	<2 ☐	2-7 ☐	>7 ☐
9. How many servings of legumes (beans, black eyed peas) do you consume per week? (1 serving: 1 cup)	<1 ☐	1-3 ☐	>3 ☐
10. How many servings of fish or shellfish do you consume per week? (1 serving: 2-3 ounces of fish or 3 ounces of shellfish)	<1 ☐	1-3 ☐	>3 ☐
11. How many times per week do you consume commercial sweets or pastries (not homemade), such as cakes, cookies, biscuits, or custard?	<3 ☐	3-5 ☐	>5 ☐
12. How many servings of nuts (including peanuts) do you consume per week? (1 serving: ¼ cup)	<1 ☐	1-3 ☐	>3 ☐
13. Do you preferentially consume chicken, turkey, or rabbit meat instead of veal, pork, hamburger, or sausage? Are you a vegetarian or vegan? ☐Yes ☐No	Yes ☐	No ☐	
14. How many times per week do you consume boiled vegetables, pasta, rice, or other dishes with a sauce of tomato, garlic, onion, or leeks without meat sautéed in olive oil?	<1 ☐	1-2 ☐	>2 ☐

Table S2: Stage of changes questions and benefits and barriers section

The next set of questions and responses are based on your knowledge, attitudes, and beliefs about a Mediterranean-based diet.

In this survey a Mediterranean-based diet is characterized by a high intake of fruit, vegetables, olive oil, nuts, and cereals; a moderate intake of fish and poultry; a low intake of dairy products, red meat, processed meats, and sweets; and wine in moderation, consumed with meals.

Check the box that applies.

1. Have you ever heard of about a Mediterranean-based diet?
   Yes [if you checked Yes, go to Question 2]
   No
2. Are you currently eating a Mediterranean-based diet?
   Yes
   No [if you checked No, go to Question 3]
3. Which best describes your thoughts about eating a Mediterranean-based diet?
   I've never thought about it.
   I'm undecided about it.
   I've decided I don't want to eat it.
   I've decided I do want to eat it.

For next set of questions check the box to indicate whether you agree or disagree with the statements below.

		Strongly Disagree	Disagree	Neither Agree or Disagree	Agree	Strongly Agree
4.	I need more information about a Mediterranean-based diet.					
5.	I do not think about the nutritional aspects of the types of foods I eat.					
6.	I find there are a lot of conflicting messages concerning healthy eating.					
7.	It would be too expensive to eat Mediterranean-based diet foods.					
8.	My family/partner won't eat a Mediterranean-based diet.					
9.	Mediterranean-based diet meals or snacks are not available when I eat out.					
10.	Someone else decides on most of the foods I eat.					
11.	It takes too long to prepare Mediterranean-based diet meals.					
12.	I don't want to change my eating habit or routine.					
13.	I don't have enough willpower to eat a Mediterranean-based diet.					
14.	I don't know how to prepare Mediterranean-based diet meals.					
15.	A Mediterranean-based diet would not be tasty enough.					
16.	There is not enough protein in a Mediterranean-based diet.					
17.	If I eat a Mediterranean-based diet, it would not be filling enough.					

18.	If I eat a Mediterranean-based diet, I would miss eating lots of junk.					
19.	There is not enough iron in a Mediterranean-based diet.					
20.	If I eat a Mediterranean-based diet, I would be worried about my health.					
21.	If I eat a Mediterranean-based diet, I wouldn't get enough energy or strength.					

For next set of responses, check the box to indicate whether you agree or disagree with the completion of the following sentence:

By eating a Mediterranean-based diet, I will …

		Strongly Disagree	Disagree	Neither Agree or Disagree	Agree	Strongly Agree
22.	Decrease my saturated fat intake					
23.	Improve my digestion					
24.	Be fit					
25.	Have a better quality of life					
26.	Live longer					
27.	Be healthier by decreasing my intake of chemicals, steroids, and antibiotics that are found in meat					
28.	Eat more fruits and vegetables					
29.	Reduce my chances of developing major diseases					
30.	Eat high protein foods					
31.	Eat foods high in fiber and roughage					
32.	Eat foods to help me control my weight					
33.	Use olive oil which is more healthy for me and/or my family					
34.	Help the environment					
35.	Help animal welfare / rights					
36.	Eat foods that contains natural ingredients					
37.	Eat foods that are easy to plan, buy, and prepare					
38.	Eat foods that are familiar					
39.	Eat foods that are like the foods I ate when I was a child					
40.	Save money					
41.	Eat foods that are good value for money					
42.	foods that tastes better than processed foods					
43.	Use olive oil to improve the taste of cooked meals					
44.	Eat a greater variety of foods					
45.	Be more content with myself					
46.	Eat foods to help me cope with stress					
47.	Eat foods to make me feel good					

Table S3: Barrier Questions with Factors

**Barrier**	*Source*
Knowledge	
I need more information about a Mediterranean-based diet.	Lea, Crawford, Worlsey, 2006 (23)
I do not think about the nutritional aspects of the types of foods I eat.	Pettinger, Holdsworth, Gerber, 2004 (24)
I find there are a lot of conflicting messages concerning healthy eating.	Pettinger, Holdsworth, Gerber, 2004
I don't know how to prepare Mediterranean-based diet meals.	Lea, Crawford, Worlsey, 2006
Convenience	
My family/partner won't eat a Mediterranean-based diet.	Lea, Crawford, Worlsey, 2006
Mediterranean-based diet meals or snacks are not available when I eat out.	Lea, Crawford, Worlsey, 2006
Someone else decides on most of the foods I eat.	Lea, Crawford, Worlsey, 2006
It takes too long to prepare Mediterranean-based diet meals.	Lea, Crawford, Worlsey, 2006
Sensory Appeal	
A Mediterranean-based diet would not be tasty enough.	Lea, Crawford, Worlsey, 2006
If I eat a Mediterranean-based diet, it would not be filling enough.	Lea, Crawford, Worlsey, 2006
If I eat a Mediterranean-based diet, I would miss eating lots of junk.	Lea, Crawford, Worlsey, 2006
Health	
There is not enough protein in a Mediterranean-based diet.	Lea, Crawford, Worlsey, 2006
There is not enough iron in a Mediterranean-based diet.	Lea, Crawford, Worlsey, 2006
If I eat a Mediterranean-based diet, I would be worried about my health.	Lea, Crawford, Worlsey, 2006
If I eat a Mediterranean-based diet, I wouldn’t get enough energy or strength.*	Lea, Crawford, Worlsey, 2006

* - Question removed from analysis due to poor internal validity of the Health factor

Table S4: Benefit Questions with Factors

**Barrier**	*Source*
Knowledge	
Decrease my saturated fat intake	Lea, Crawford, Worlsey, 2006 (23)
Improve my digestion	Lea, Crawford, Worlsey, 2006
Have a better quality of life	Lea, Crawford, Worlsey, 2006
Live longer	Lea & Worsley, 2002 (25)
Eat more fruits and vegetables	Lea & Worsley, 2002
Reduce my chances of developing major diseases	Harnack, Block, Subar, Lane, Brand, 1997 (15)
Eat high protein foods	Steptoe, Pollard, Wardle, 1995 (27)
Eat foods high in fiber and roughage	Steptoe, Pollard, Wardle, 1995
Use olive oil which is more healthy for me and/or my family	Thompson, Haziris, Alekos, 1994 (26)
Eat a greater variety of foods	Lea, Crawford, Worlsey, 2006
Weight Loss	
Be fit	Lea, Crawford, Worlsey, 2006
Eat foods to help me control my weight	Lea, Crawford, Worlsey, 2006
Natural Content	
Be healthier by decreasing my intake of chemicals, steroids, and antibiotics that are found in meat	Lea & Worsley, 2002
Eat foods that contains natural ingredients	Lea, Crawford, Worlsey, 2006
Ethical Concerns	
Help the environment	Lea, Crawford, Worlsey, 2006
Help animal welfare / rights	Lea, Crawford, Worlsey, 2006
Convenience	
Eat foods that are easy to plan, buy, and prepare	Pieniak, Verbeke, Vanhonacker Guerrerno, Hersleth, 2009 (28)
Familiarity	
Eat foods that are familiar	Steptoe, Pollard, Wardle, 1995
Eat foods that are like the foods I ate when I was a child	Steptoe, Pollard, Wardle, 1995
Price	
Save money	Lea, Crawford, Worlsey, 2006
Eat foods that are good value for money	Steptoe, Pollard, Wardle, 1995
Sensory Appeal	
Eat foods that tastes better than processed foods	Pettinger, Holdsworth, Gerber, 2004 (24)
Use olive oil to improve the taste of cooked meals	Thompson, Haziris, Alekos, 1994
Mood	
Be more content with myself	Lea, Crawford, Worsley, 2006
Eat foods to help me cope with stress	Steptoe, Pollard, Wardle, 1995
Eat foods to make me feel good	Steptoe, Pollard, Wardle, 1995

Table S5: Demographic and anthropomorphic questions

Finally, we would like to ask you a few questions about yourself

1. Are you male or female?
   a) Male ☐
   b) Female ☐

2. How old are you?
   a) less than 18 ☐
   b) 18-24 ☐
   c) 25-34 ☐
   d) 35-44 ☐
   e) 45-54 ☐
   f) 55-64 ☐
   g) 65-74 ☐
   h) more than 75 ☐

3. What is your ethnic origin?
   a) White ☐
   b) Black African ☐
   c) Black other ☐
   d) Indian ☐
   e) Pakistani ☐
   f) Chinese ☐
   g) Asian- other ☐
   Please specify:………………………………………
   h) Any other ethnic group ☐
   Please specify:………………………………………

4. What is the highest level of education you have completed?
   a) Elementary school ☐
   b) Middle school ☐
   c) High school diploma ☐
   d) GED ☐
   e) Technical or trade certificate ☐
   f) Associate degree ☐
   g) Bachelor's degree ☐
   h) Master's or professional degree ☐

5. Do you have any health or nutrition related qualifications?
   a) Yes ☐
   Please specify: ………………………………………
   b) No ☐

6. What is your body weight?
   Please specify: ………………………………………

7. What is your height?
   Please specify: ………………………………………
